# Respiratory infections and chronic cough due to triple A (Allgrove) syndrome

**DOI:** 10.1002/ccr3.2683

**Published:** 2020-01-29

**Authors:** Hengqi Zheng, Ramesh S. Iyer, M. Cristina Pacheco, Jennifer Jean Soares, Kaalan Johnson, Mary Len, Lusine Ambartsumyan

**Affiliations:** ^1^ Division of Gastroenterology Seattle Children's Hospital Seattle Washington; ^2^ Department of Radiology Seattle Children's Hospital Seattle Washington; ^3^ Department of Laboratories Seattle Children's Hospital Seattle Washington; ^4^ Division of Pulmonology Seattle Children's Hospital Seattle Washington; ^5^ Department of Otolaryngology Seattle Children's Hospital Seattle Washington

**Keywords:** achalasia, Allgrove syndrome, chronic cough, dysphagia

## Abstract

Cough and respiratory infections are common in pediatrics. Our case report illustrates the need for pediatricians to consider rare diagnoses such as genetic syndromes and primary gastrointestinal motility disorders in patients with unremitting respiratory and gastrointestinal symptoms. Early identification provides early intervention and reduces long‐term morbidity for pediatric patients.

## Graphical abstract

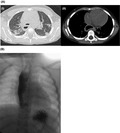

## 1 INTRODUCTION

Cough and respiratory infections are common conditions in pediatrics. The differential diagnoses for both are broad, encompassing respiratory conditions such as asthma to extrapulmonary conditions such as gastroesophageal reflux disease (GERD). While GERD has long been implicated as a cause of lung disease in children, gastrointestinal dysmotility such as achalasia should also be included in the differential diagnosis.^1^ Achalasia is characterized by absent peristalsis of the esophagus and abnormal relaxation of the lower esophageal sphincter (LES) and is associated with several genetic syndromes such as Triple A syndrome.^2^

## 2 CASE REPORT

We present a 3‐year‐old female with multiple respiratory infections starting at the age of 10 months, necessitating multiple hospital admissions with intensive care unit stay for virus bronchiolitis and bacterial pneumonia. At 2 years of age, she developed episodes of nonbloody, nonbilious emesis with ingestion of liquids and solids. However, she continued to take solids and liquids of all consistencies without gagging, choking, or coughing paired with normal growth and development. Her mother denied noisy breathing, wheezing, or snoring. Of note, her mother did mention that the patient has never had tears with either emotional or painful stimulation.

The patient was evaluated by pediatric pulmonology, chest clinic, gastroenterology, neurology, immunology, and otolaryngology and underwent a series of laboratory tests, radiographic examinations, and procedures. She was trialed on beclomethasone dipropionate hydroalkane (HFA) 40 microgram (mcg) two times a day, levalbuterol 4 puffs every 4 hours as needed, and required courses of steroids with mild intermittent improvements in respiratory symptoms. She was also trialed on lansoprazole 1 milligram per kilogram per dose (mg/kg/dose) two times a day 15‐30 minutes before meals, cyproheptadine 0.25 mg/kg/dose every night at bedtime, and erythromycin 10 mg/kg/dose two times a day with no improvement in emesis or reflux.

**Figure 1:**
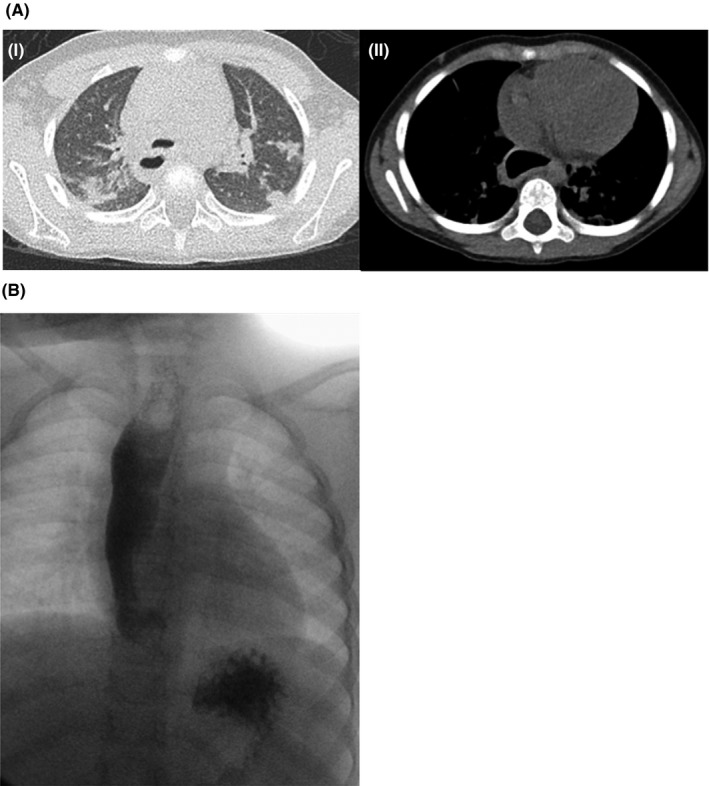
A, Noncontrast HRCT at the time of diagnosis showing (i) bilateral polylobar scattered airspace consolidation and ground glass opacities, particularly within the upper lobes, and (ii) a patulous thoracic esophagus, greatest in the distal segment proximal to the GEJ. B, Fluoroscopic esophagram showing a dilated esophagus with evidence of poor clearance and dysmotility for liquid contrast. A patent GEJ was never well visualized, with a small amount of contrast present in stomach

**Figure 2:**
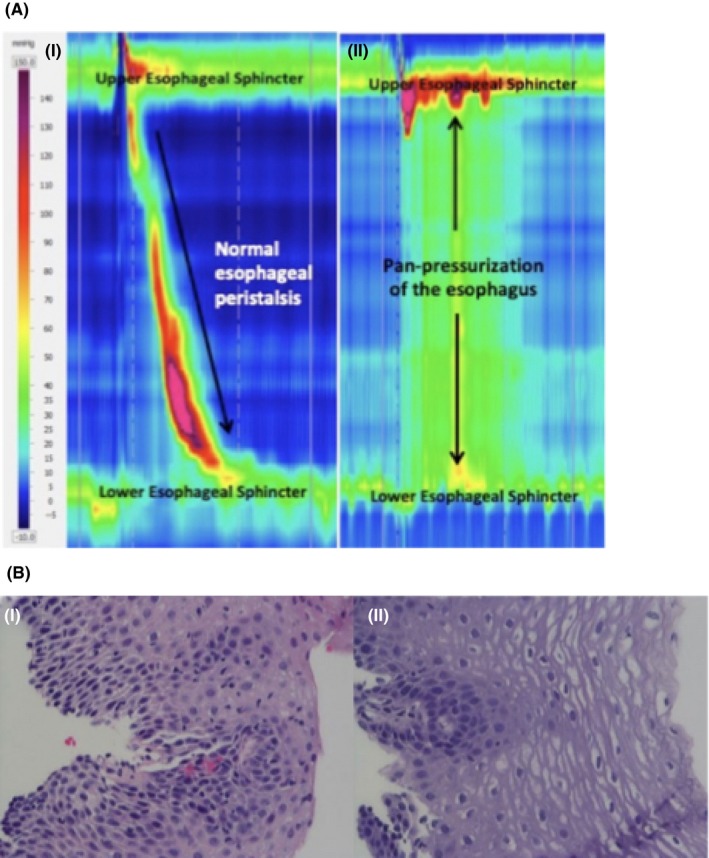
A, High‐resolution esophageal manometry (HREM) with impedance: (i) Normal peristalsis and relaxation of the lower esophageal sphincter (ii) absent peristalsis, absent lower esophageal sphincter relaxation, and pan‐pressurization of the esophagus consistent with type 2 Achalasia. B, Hematoxylin and eosin staining at 400× of distal esophagus (i) original esophageal biopsy showing increased peripapillary lymphocytes and spongiosis of the squamous mucosa; (ii) showing complete resolution on follow‐up 4 mo after original biopsy

A high‐resolution computed tomography scan (HRCT) revealed scattered airspace consolidation and ground glass opacities, and a patulous thoracic esophagus. (Figure 1A) The patient underwent a fluoroscopic esophagram showing a dilated esophagus and a closed gastroesophageal junction (GEJ) with evidence of poor clearance of liquid contrast (Figure 1B). She underwent triple endoscopy with (a) flexible bronchoscopy which showed no marked tracheomalacia without laryngomalacia, and moderately edematous respiratory mucosa, and bronchoalveolar lavage fluid which returned normal respiratory flora on cultures; (b) microdirect laryngoscopy and rigid bronchoscopy which showed a normal supraglottis, glottis, interarytenoid anatomy, and trachea beyond the tracheomalacia without evidence of tracheoesophageal fistula or laryngeal cleft; and (c) esophagogastroduodenoscopy (EGD) revealing normal esophageal mucosa with dilation. EGD biopsies revealed spongiosis with increased peripapillary lymphocytes of unclear etiology in the esophagus (Figure 2Bi); after treatment with proton‐pump inhibitors, repeat EGD 4 months later showed normalization of esophageal histologic findings (Figure 2Bii). She then underwent high‐resolution esophageal manometry (HREM) which demonstrated absent peristalsis, absent LES relaxation, and pan‐pressurization of the esophagus consistent with type 2 Achalasia (Figure 2Aii).

Over time, she was noted to have increasing weakness, low energy, and a new tan pigmentation to her skin. She was then evaluated by pediatric endocrinology, diagnosed with adrenal insufficiency resistant to adrenocorticotropic hormone (ACTH), and started on hydrocortisone 5 mg three times per day and fludrocortisone 0.1 mg each morning.

She underwent surgical correction of achalasia with a laparoscopic Heller myotomy and Dor fundoplication and discharged home tolerating full feeds by mouth without emesis. Genetic consultation was then obtained and testing revealed compound heterozygosity for two variants of AAAS gene–c.43 C > A and c1066_1067delCT; thereby confirming the diagnosis of Triple A syndrome.

## 3 DISCUSSION

Triple A or Allgrove syndrome is a rare diagnosis characterized by autosomal recessive inheritance with symptoms of ACTH‐resistant adrenal insufficiency, alacrima, and achalasia. This syndrome stems from mutations of the AAAS gene on chromosome 12q13 leading which codes for the ALADIN nuclear pore scaffolding protein. The syndrome commonly presents in the first few years of life with hypoglycemia from adrenocortical insufficiency or with achalasia. However, presentations can be varied with neurophysiologic and autonomic abnormalities, and adult‐onset cases have been identified. Our case is unique in that the presenting symptoms were respiratory in nature with cough and respiratory infections.

The clinical presentation of achalasia is variable in pediatric patients and symptoms may be insidious and are commonly attributed to GERD. Younger children may present with respiratory symptoms such as chronic diurnal cough, choking, and recurrent respiratory infections and aspiration pneumonias.^3^

A fluid‐filled esophagus on chest x‐ray and/or a dilated esophagus on HRCT (Figure 1A) such as in our patient in the setting of chronic lung disease is concerning for esophageal dysmotility and warrants further investigation. Pulmonary function findings of obstructive and restrictive disease, and HRCT findings of tracheal‐bronchial compression and parenchymal lesions (ground glass opacities, fibrosis, bronchiectasis) were reported in adult achalasia patients. Lung involvement was thought to be secondary to extrinsic compression and recurrent microaspiration. Improvement in respiratory symptoms, pulmonary function on PFTs and parenchymal changes on HRCT have been demonstrated in adults following treatment with Heller myotomy or pneumatic dilation. Our patient had a complete resolution of her respiratory and gastrointestinal symptoms following surgical intervention.

Esophagram findings concerning for achalasia include esophageal dilation, delayed emptying of contrast, and a narrow GEJ classically referred to as having a “bird‐beak” appearance. An upper endoscopy demonstrates a lack of stricture and may reveal a dilated esophagus with retained saliva and food products. However, it is important to ensure that there is no anatomical or primary mucosal abnormality that can mimic esophageal dysmotility. In our case, most likely, the increased lymphocytes were present due to residual food in the esophagus supported by the known association of nonachalasia‐related motility disorders with lymphocytic esophagitis.^4^

The gold standard test for diagnosis of esophageal achalasia is HREM where the absence of esophageal peristalsis ± abnormal LES relaxation is diagnostic of achalasia. Treatments, such as esophageal pneumatic balloon dilation and surgical myotomy, alleviate the functional obstruction at the LES and facilitate esophageal emptying. 

While there is no definitive treatment for Triple A syndrome, recognition of symptoms, timely diagnosis, and the treatment of the individual components of the syndrome is crucial for patient prognosis. Our case highlights the need to consider esophageal motility disorders such as achalasia in patients presenting with refractory symptoms that are normally attributed to respiratory problems or gastroesophageal reflux.

## CONFLICT OF INTEREST

The authors have indicated they have no potential conflicts of interest to disclose.

## AUTHOR CONTRIBUTIONS

Hengqi (Betty) Zheng: Dr Zheng contributed to the conception, collected the clinical data, drafted the initial manuscript, reviewed and critically revised the manuscript for important concepts, and approved the final manuscript as submitted. Ramesh S. Iyer, M. Cristina Pacheco, Lusine Ambartsumyan: Dr Iyer, Dr Pacheco, and Dr Ambartsumyan collected clinical data, critically revised the manuscript for important concepts, and approved the final manuscript as submitted. Jennifer Jean Soares, Kalaan Johnson, Mary Len: Dr Soares, Dr Johnson, and Dr Len collected clinical data, critically revised the manuscript t for important concepts, and approved the final manuscript as submitted. All authors approved the final manuscript as submitted and agree to be accountable for all aspects of the work.
